# Nerve growth factor regulates endothelial cell survival and pathological retinal angiogenesis

**DOI:** 10.1111/jcmm.14002

**Published:** 2019-01-24

**Authors:** Maria Troullinaki, Vasileia‐Ismini Alexaki, Ioannis Mitroulis, Anke Witt, Anne Klotzsche–von Ameln, Kyoung‐Jin Chung, Triantafyllos Chavakis, Matina Economopoulou

**Affiliations:** ^1^ Institute of Clinical Chemistry and Laboratory Medicine University Clinic Carl Gustav Carus, TU Dresden Dresden Germany; ^2^ Department of Ophthalmology University Clinic Carl Gustav Carus, TU Dresden Dresden Germany

**Keywords:** angiogenesis, apoptosis, endothelium, hypoxia, mitochondria, NGF, retinopathy

## Abstract

The mechanism underlying vasoproliferative retinopathies like retinopathy of prematurity (ROP) is hypoxia‐triggered neovascularisation. Nerve growth factor (NGF), a neurotrophin supporting survival and differentiation of neuronal cells may also regulate endothelial cell functions. Here we studied the role of NGF in pathological retinal angiogenesis in the course of the ROP mouse model. Topical application of NGF enhanced while intraocular injections of anti‐NGF neutralizing antibody reduced pathological retinal vascularization in mice subjected to the ROP model. The pro‐angiogenic effect of NGF in the retina was mediated by inhibition of retinal endothelial cell apoptosis. In vitro, NGF decreased the intrinsic (mitochondria‐dependent) apoptosis in hypoxia‐treated human retinal microvascular endothelial cells and preserved the mitochondrial membrane potential. The anti‐apoptotic effect of NGF was associated with increased BCL2 and reduced BAX, as well as with enhanced ERK and AKT phosphorylation, and was abolished by inhibition of the AKT pathway. Our findings reveal an anti‐apoptotic role of NGF in the hypoxic retinal endothelium, which is involved in promoting pathological retinal vascularization, thereby pointing to NGF as a potential target for proliferative retinopathies.

## 1 INTRODUCTION

Proliferative retinopathies like diabetic retinopathy (DR) or retinopathy of prematurity (ROP) are major causes of blindness and pose a major therapeutic challenge. In these pathologies, hypoxia‐induced pro‐angiogenic factors lead to pathological neovascularization, which may compromise neuronal function in the retina.^1^, ^2^ Current treatments target the exuberant vascular endothelial growth factor (VEGF)‐induced vasoproliferation, aiming at reducing the vascular leakage and haemorrhage resulting from the newly formed ectopic vessels.^1^ However, anti‐VEGF treatment may deprive neuronal retinal cells of the neuroprotective actions of VEGF.^3^ Furthermore, DR displays significant neurodegeneration already before any vascular alterations, which has resulted in a debate about the potential therapeutic use of neuroprotective agents in retinopathies.^4^, ^5^ Therefore, it is important to elucidate how naturally occurring neuroprotective agents may influence the endothelium in the context of proliferative retinopathies.

The neurotrophin nerve growth factor (NGF) is a key player in survival, growth and differentiation of neuronal cells.^6^ Bioactive NGF derives from a precursor molecule, called proNGF,^7^ which is processed and cleaved either intracellularly^8^ or extracellularly by plasmin and matrix metalloproteinases.^9^ NGF binds with high affinity to the Tropomyosin receptor kinase A (TrkA), a receptor with tyrosine kinase activity.^6^ Upon NGF binding, TrkA dimerizes and is auto‐phosphorylated at its intracellular part,^6^, ^10^ resulting in activation of signalling pathways, such as the Ras‐Raf‐MEK‐ERK pathway and the PI3K‐AKT pathway, which are involved in neuronal survival.^6^, ^11^ NGF can also bind with lower affinity to the p75 receptor, which induces the JNK or the NFκB signalling pathway through direct interaction with intermediates, such as RhoA or TRAF proteins.^6^, ^11^

The neuroprotective effect of NGF has also been studied in various neurodegenerative diseases of the eye.^12^ Phase 1/2 and pilot clinical trials have used NGF eye‐drops in an attempt to rescue or reduce the degeneration of retinal neuronal or ganglion cells in retinitis pigmentosa and glaucoma patients.^12^, ^13^, ^14^ In addition, patients with DR have increased NGF concentration in the vitreous, as compared to non‐diabetic individuals.^4^

Besides neuronal cells, NGF may act on immune cells or endothelial cells (EC).^15^, ^16^, ^17^, ^18^, ^19^ For instance, NGF prevents mast cell apoptosis,^20^ while it also induces mast cell activation through its interaction with platelets, which in turn induce tissue apoptosis in ischemic stroke.^21^, ^22^ Endothelial cells express NGF receptors.^17^, ^18^, ^19^ NGF activates through TrkA the PI3K/AKT, MEK/ERK and PLCγ/PKC pathways^17^, ^18^, ^23^ and may exert pro‐angiogenic actions.^19^, ^24^ For instance, NGF enhanced the proliferation of brain capillary EC^19^ and of human umbilical vein EC and promoted angiogenesis in chorioallantoic membranes of chicken embryos.^17^ Furthermore, NGF promoted vascularization in hindlimb ischemia^25^ as well as tumor angiogenesis.^26^

These observations prompted us to investigate the effects of NGF in the environment of the retina, where neuronal and EC display close spatial and functional interactions. We studied the role of NGF in the context of proliferative retinopathies by using the mouse ROP model. We found a pro‐angiogenic effect of NGF, which was associated with downregulation of retinal EC apoptosis and with preservation of mitochondrial membrane potential especially under hypoxic conditions. Our findings therefore indicate that NGF may represent a potential target for proliferative retinopathies.

## 2 MATERIALS AND METHODS

### 2.1 Mice and ROP model

Wild type C57BL/6 mice were obtained from Janvier Labs (Le Genest‐Saint‐Isle, France). Mice were subjected to the ROP model as described.^27^, ^28^, ^29^ Briefly, 7–day old pups were exposed for 5 days to hyperoxia (75% O_2_) together with their nursing mothers. Thereafter, pups and mothers were returned to room air (21% O_2_). On postnatal day 14 (p14), anesthetized pups received an intraocular injection of anti‐NGF antibody (1 μg/eye; Abcam, UK) in the right eye and the same amount of control rabbit IgG (Abcam) in the left eye; injections were performed under a stereoscope (Stemi C‐2000; Zeiss, Oberkochen, Germany), as described.^30^ In other experiments, pups received from p13 to p16 twice per day eye drops of NGF (Merck Millipore, Darmstadt, Germany) (3 μL, 200 μg/mL) in the right eye and same volume of PBS in the left eye, as described.^31^ At p17, pups were killed and eyes were retrieved for further analysis of the retinas.^32^ Animal experiments were approved by the Landesdirektion Sachsen, Germany.

### 2.2 Periodic acid – Schiff staining

For assessment of abnormal retinal angiogenesis, eyes were isolated at p17 from pups subjected to the ROP model and were fixed overnight in 4% PFA at 4°C. Paraffin‐embedded sections (4 μm) that included the head of the optic nerve were prepared. After de‐paraffinization of retinal sections by overnight incubation at 60°C and subsequent incubation in Roticlear^®^ solution (Carl Roth, Karlsruhe, Germany), serial washes with ethanol followed. Sections were washed again with PBS and tap water and they were stained by immersing the slides first in periodic acid (VWR Chemicals Prolab, Dresden, Germany) and then in Schiff reagent (SAV‐liquid production GmbH, Flintsbach, Germany) followed by washes with PBS and staining with Hematoxylin (SAV‐liquid production GmbH). Neovascular cell nuclei anterior to the internal limiting membrane were counted in each retinal section and the mean of at least 10 counted sections was calculated per retina.^32^ Moreover, images were acquired with a computerized microscope (Zeiss) and analysed with the ZEN software (Zen Software Ltd, Rochdale, UK).

### 2.3 Apoptosis assay in retinas

Eyes were isolated from pups at p17, snap‐frozen and embedded in OCT compound (Tissue‐Tek, CA, USA). Frozen serial sections (10 μm) were obtained and kept at −20°C until further processing. For visualization of apoptotic EC in the retina, sections were fixed for 10 minutes with acetone at −20°C. The slides were left to dry at room temperature and after three washes with PBS, they were permeabilized and blocked for 1 hour in PBS with 3% goat serum/0.2% Triton X‐100/5% BSA followed by overnight incubation with a rabbit anti‐cleaved caspase‐3 antibody (1:250; Cell Signaling Technology, Inc., Danvers, MA, USA). On the next day, sections were washed with PBS and incubated for 1 hour with Alexa Fluor 568‐conjugated secondary goat anti‐rabbit antibody (Life Technologies, Darmstadt, Germany), with FITC‐conjugated Isolectin GS IB4 (Life Technologies) to stain for the vascular endothelium and with DAPI (1:10 000; Life Technologies) at room temperature. The cleaved caspase 3‐positive vascular cells were counted in each section and at least 10 sections/eye were evaluated. Images were acquired with a Zeiss microscope (Zeiss, Jena, Germany) and were analysed with the AxioVision software (Zeiss).^32^, ^33^, ^34^

### 2.4 Cell culture and in vitro treatments for Western Blot analysis

Human retinal microvascular endothelial cells (HRMEC) were purchased from Cell‐Systems (Kirkland, WA, USA). Cells were cultured in endothelial growth medium (EGM; PromoCell, Heidelberg, Germany) with SupplementMix (PromoCell) at 37°C and 5% CO_2_ and seeded in culture dishes pre‐coated with 0.2% gelatin (Sigma‐Aldrich, Munich, Germany).

For the experiments regarding BCL2 and BAX protein expression, cells were treated with NGF (100 ng/mL; Merck Millipore) in starvation medium (plain EGM without supplement) for 3.5 hours under hypoxia (1% O_2_). For studying the ERK and AKT signaling pathway, HRMEC were cultured to confluence and exposed overnight to hypoxia in EGM without SupplementMix with 1% FBS; thereafter, cells were treated with 100 ng/mL NGF in plain EGM with 1% FBS and analysed at different time points under hypoxic conditions (1% O_2_), as indicated in the figures.

Western blot analysis was performed as previously described.^16^, ^33^ After the aforementioned treatments, cells were washed twice with ice‐cold PBS and lysed in RIPA buffer (Santa Cruz, Heidelberg, Germany) including phosphatase and protein inhibitors (Roche, Mannheim, Germany) and benzonase (Sigma‐Aldrich). Thirty μg of protein were boiled at 94°C for 4 minutes, loaded onto 4%‐12% gradient SDS gels (Nupage, Invitrogen, Waltham, MA, USA) and then transferred onto 0.2 μm pore‐sized Hybond nitrocellulose membranes (Amersham Biosciences, Germany). Membranes were blocked for 1 hour in 5% non‐fat milk diluted in TBS‐T buffer (0.15 M NaCl, 2.7 mmol/L KCl, 24.8 mmol/L Trisbase, 0.1% Tween‐20), and immunoblotted overnight at 4°C. Antibodies used were mouse anti‐BCL2 (1:500; Cell Signaling), rabbit anti‐BAX (1:500; Cell Signaling), rabbit anti‐phosphoAKT (1:500; Cell Signaling), rabbit anti‐AKT (1:500; Cell Signaling), rabbit anti‐phosphoERK (1:500; Cell Signaling), rabbit anti‐ERK (1:500; Cell Signaling) and rabbit anti‐Vinculin (1:500; Cell Signaling). Anti‐mouse and anti‐rabbit HRP‐conjugated secondary antibodies (R&D, Wiesbaden‐Nordenstadt, Germany) were used at a dilution of 1:2000. After washes with TBS‐T, membranes were developed using SuperSignal West Pico Chemiluminescent Substrate (Life Technologies) or SuperSignal West Fempto Chemiluminescent Substrate (Life Technologies) and the luminescent image analyser LAS‐3000 (Fujifilm, Dusseldorf, Germany). The intensity of the bands was quantified with the Fiji software.^35^

### 2.5 In vitro apoptosis assay

HRMEC were cultured on gelatin‐coated coverslips placed in 24‐well‐ or 96‐well plates until confluence. On the next day, the medium was changed into plain EGM and cells were exposed to normoxia (21% O_2_) or hypoxia (1% O_2_) for 5 hours, followed by medium change to plain EGM with 100 ng/mL NGF or vehicle control and incubation for further 20 hours in normoxia or hypoxia. For the inhibition of the AKT pathway, Wortmannin (10 ng/mL; R&D Systems) was added in plain EGM 1 hour before the aforementioned 25 hour‐treatment. After washing, cells were fixed with 4% PFA for 15 minutes at room temperature followed by permeabilization and blocking in PBS including 5% goat serum and 0.3% Triton X‐100 for 2 hours and overnight incubation with rabbit cleaved caspase‐3 antibody (1:250; Cell Signaling Technology, Inc.) and FITC‐conjugated Isolectin GS IB4 (1:100; Life Technologies). After washings, cells were incubated for 1 hour with secondary Alexa Fluor 568‐conjugated anti‐rabbit antibody (1:350; Life Technologies) and DAPI (1:10 000) for nuclei visualization at room temperature. Random images were taken with an inverted fluorescence microscope (Zeiss, Oberkochen) (20×). More than 450 cells per condition were analysed in a blinded fashion with Fiji software. The percentage of cleaved caspase 3‐positive cells over the total cell number was calculated.

### 2.6 Cell proliferation assay

HRMEC were plated onto gelatin‐coated 12‐well plates at a density of 15 000 cells/well. On the next day, the medium was replaced with fresh EGM with supplementMix containing BrdU (250 ng/mL; BD Bioscience, Heidelberg, Germany) and cells were exposed for 5 hours to normoxia 21% O_2_) or hypoxia (1% O_2_) in the presence of 100 ng/mL NGF or vehicle control (PBS). Cells were then washed with PBS, detached with trypsin and fixed with cold 70% ethanol at 4°C for 30 minutes. For DNA denaturation, cells were incubated with 2 N HCl for 30 minutes at room temperature followed by neutralization with 0.1 N Na_2_B_4_O_7_ pH 8.5. Thereafter, cells were incubated with an anti‐BrdU FITC‐conjugated antibody (1:100; BD Bioscience) for 30 minutes at room temperature in the dark, washed with PBS containing 5% FBS and were analysed by FACS using a BD FACSCanto II (BD Biosciences).^36^ Data were analysed with the BD FACSDiva Version 6.1.3 software (BD Biosciences).

### 2.7 Analysis for cleaved PARP

HRMEC were seeded onto gelatin‐coated 60 mm diameter dishes. On the next day, they were treated for 24 hours with PBS or 100 ng/mL NGF in plain EGM under normoxia (21% O_2_) or hypoxia (1% O_2_). The samples were washed twice with cold PBS and lysed in RIPA Buffer (Santa Cruz, Heidelberg, Germany) including phosphatase, protein inhibitors (Roche, Mannheim, Germany) and benzonase (Sigma‐Aldrich). Lysates were collected and protein concentration was determined with the BCA Protein Assay kit (Invitrogen). Cleaved PARP levels were studied in equal amounts of protein lysates with the Apoptosis Whole Cell Lysate Kit (Meso Scale Diagnostics, Rockville, MD, USA). Samples were then analysed in a plate reader (QuickPlex SQ 120; Meso Scale Diagnostics) following the manufacturer's protocol.

### 2.8 Mitochondrial load and ΔΨm measurement

For ∆Ψm measurement, HRMEC were stained with the TMRE dye (Abcam) according to manufacturer's instructions. Briefly, cells were cultivated on gelatin pre‐coated 96‐well plates for 1 day and they were then treated for 3.5 hours under hypoxia (1% O_2_) with PBS or NGF (100 ng/mL) in plain EGM. Cells were then incubated for 30 minutes with TMRE (500 nmol/L; Abcam) diluted in PBS at 37°C under hypoxia. After washing with pre‐warmed PBS, cells were immediately analysed in a fluorescence plate reader (Ex/Em: 549/575 nm).

To assess the mitochondrial load, HRMEC were seeded onto gelatin pre‐coated 12‐well plates. On the next day, cells were treated for 3.5 hours under hypoxic conditions in the presence of NGF (100 ng/mL) or PBS in plain EGM. Cells were then washed once with pre‐heated HBSS including Ca^2+^/Mg^2+^ and stained for 30 minutes with 20 nmol/L MitoTracker Green FM probe (Life Technologies) diluted in pre‐warmed HBSS including Ca^2+^/Mg^2+^ at 37°C in the dark. After washes with pre‐warmed HBSS including Ca^2+^/Mg^2+^, cells were analysed immediately by flow cytometry using a BD FACSCanto II and the BD FACSDiva Version 6.1.3 software.

### 2.9 Statistical analysis

All values are presented as the mean ± SEM. Statistical analysis was performed by Mann–Whitney *U* test or Student's *t* test with *P* ≤ 0.05 as a significance level using GraphPad Prism 6.0 Software (GraphPad Software, CA, USA).

## 3 RESULTS

### 3.1 NGF increased pathological neovascularization in mice subjected to ROP model by inhibiting EC apoptosis

**Figure 1:**
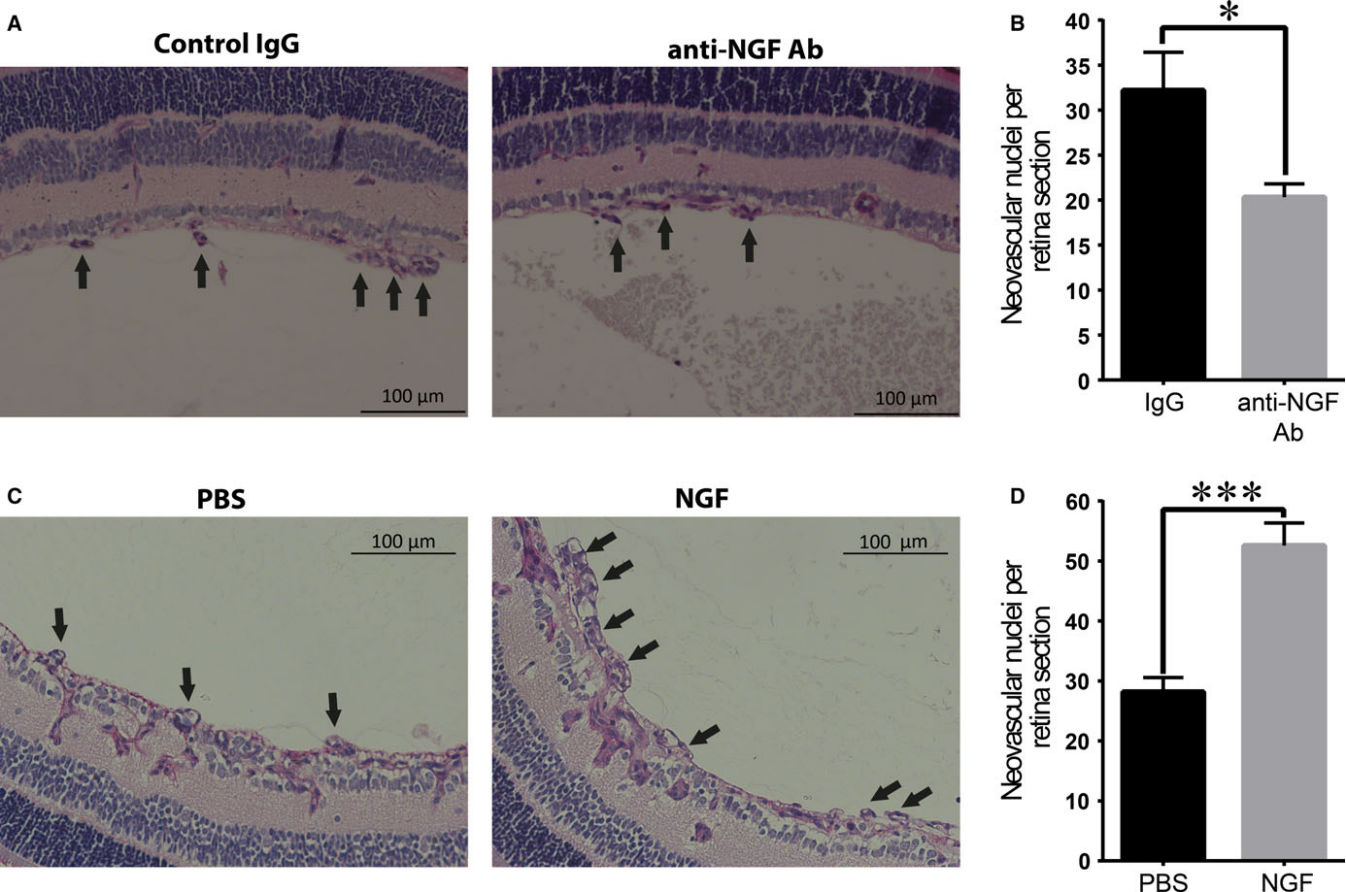
NGF promotes pathological angiogenesis in mice subjected to ROP. (A, B) C57BL/6 pups were subjected to the ROP model. At day p14 they received anti‐NGF antibody or control IgG intraocularly. At day p17, pups were killed, and neovascularization in Periodic acid – Schiff (PAS)‐stained retinal cross‐sections was quantified. (A) Representative images of PAS stained retinal cross‐sections, neovascular tufts are depicted by arrows; scale bar: 100 μm. (B) Quantification of pathological angiogenesis was performed by counting the nuclei of vessels anterior to the inner limiting membrane. The number of neovascular nuclei per retinal section is shown. Data are presented as mean ± SEM (n = 15 eyes/group); *P ≤ 0.05. (C, D) C57BL/6 mice were subjected to the ROP model. From p13 to p16 they received twice per day NGF eye drops in the right eye and vehicle control (PBS) in the left eye. At day p17, pups were killed and neovascularization was quantified in PAS‐stained retinal sections. (C) Representative images of PAS stained retinal cross‐sections, neovascular tufts are depicted by arrows; scale bar: 100 μm. (D) The number of neovascular nuclei of vessels anterior to the inner limiting membrane was quantified. Data are presented as mean ± SEM (n = 5); ***P ≤ 0.001

We initially investigated the effect of NGF on retinal neovascularization in mice subjected to the ROP model. After exposure to hyperoxia (75% O_2_) from p7 to p12, pups were returned to normoxia. Intraocular injection of anti‐NGF antibody at p14 led to a significant decrease in pathological neovascularization (neovascular tufts invading the vitreous cavity) in p17 retinas, as compared to retinas from the contralateral eye that received an injection of control IgG (Figure 1A and B). Thus, endogenous NGF promoted pathological retinal neovascularization in the ROP model.

**Figure 2:**
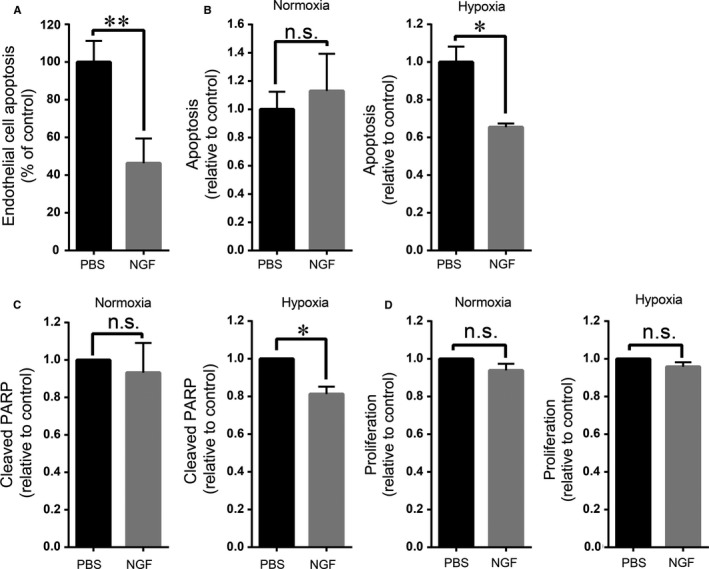
NGF reduces apoptosis of endothelial cells in the course of ROP and under hypoxic conditions in vitro. (A) Sections of p17 retinas of ROP mice that were treated with NGF eye drops or vehicle control (PBS), as described under Figure 1C and D, were analysed for endothelial cell apoptosis, as described in Section 2. The number of apoptotic endothelial cells was counted per retinal section. Endothelial cell apoptosis in the retina is presented as % of control; apoptosis in vehicle‐treated retinas was set as 100%. Data are presented as mean ± SEM (n = 7); **P ≤ 0.01. (B) HRMEC apoptosis was studied under normoxic and hypoxic conditions in the presence of vehicle control (PBS) or 100 ng/mL NGF by performing cleaved caspase‐3 staining, as described in Section 2. HRMEC apoptosis is shown relative to control; apoptosis in the presence of PBS control was set as 1. Data are presented as mean ± SEM and are from one experiment performed in triplicate; similar results were observed in at least 3 separate experiments. *P ≤ 0.05. (C) Expression of cleaved PARP was studied under normoxic and hypoxic conditions in the presence of vehicle control (PBS) or 100 ng/mL NGF, as described in Section 2. Expression of cleaved PARP is shown relative to control; expression of cleaved PARP in the presence of PBS control was set as 1 in each experiment. Data are presented as mean ± SEM (n = 4); *P ≤ 0.05. (D) HRMEC were exposed to normoxia or hypoxia in the presence of PBS or 100 ng/mL NGF and endothelial proliferation was assessed by BrdU incorporation, as described in Section 2. Proliferation is shown relative to control; proliferation in the presence of PBS was set as 1 in each experiment. Data are presented as mean ± SEM (n = 5); n.s.: not significant

To further substantiate this pro‐angiogenic effect of NGF, pups subjected to the ROP protocol received twice daily NGF eye drops on one eye and vehicle control (PBS) on the other eye from p13 to p16.^31^ A significant increase of pathological neovascularization in p17 retinas of the eyes that were treated with NGF drops was observed, as compared to the contralateral control‐treated eye (Figure 1C and D). This effect was associated with decreased apoptosis of EC in the retinas of the NGF‐treated eyes (Figure 2A). Thus, both endogenous (Figure 1A and B) as well as exogenously delivered NGF (Figure 1C and D) increased pathological neovascularization in the retina of pups subjected to the ROP model. On the contrary, NGF eye‐drop administration did not affect the revascularization of the avascular area in the same retinas (data not shown). Together, our data suggest a pro‐angiogenic effect of NGF in the context of neovascularization under hypoxic stress conditions.

### 3.2 NGF reduced apoptosis in HRMEC under hypoxic conditions

Our in vivo findings prompted us to investigate how NGF affects cell growth and survival of retinal EC under different oxygen concentrations (21% and 1% O_2_) in vitro. As an appropriate model, we chose HRMEC, EC from human retina previously shown to express TrkA.^37^ We initially examined the effect of NGF on HRMEC apoptosis induced by starvation under normoxic or hypoxic conditions.^38^, ^39^ Cells were treated with NGF or vehicle control and then they were stained for cleaved caspase‐3. Under hypoxic conditions, NGF significantly reduced the apoptosis of HRMEC, as compared to control treated cells, while it had no effect on apoptosis of cells incubated under normoxic conditions (Figure 2B). The anti‐apoptotic effect of NGF was further confirmed by the detection of cleaved PARP, which was significantly reduced by NGF treatment solely under hypoxic conditions (Figure 2C).

Next, we tested whether NGF affects HRMEC cell proliferation. To this end, cells were treated with NGF under normoxic (21% O_2_) or hypoxic (1% O_2_) conditions and proliferation was assessed by BrdU incorporation. NGF did not influence cell proliferation of HRMEC in normoxia or in hypoxia (Figure 2D). This result is in accordance with previous studies, which also showed no effect of NGF on retinal EC proliferation.^40^, ^41^ Thus, NGF reduced the apoptosis of HRMEC specifically under hypoxic conditions without affecting HRMEC proliferation.

**Figure 3:**
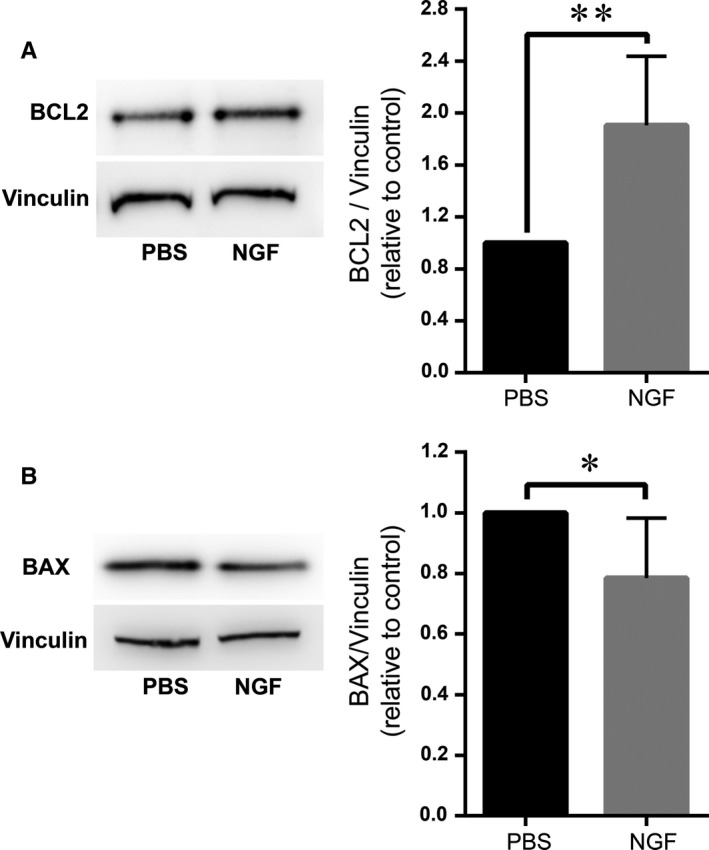
NGF regulates BCL2 and BAX protein expression. (A) Left: Representative cropped blot images showing immunoblotting for BCL2 in HRMEC treated with NGF or control (PBS) under hypoxic conditions, as described in Section 2. Vinculin was used as loading control. Right: Densitometric analysis of BCL2 immunoblotting is shown. The protein amounts of BCL2 were normalized to Vinculin and are shown relative to control; the ratio BCL2/Vinculin in HRMEC treated with PBS was set as 1 in each experiment. Data are presented as mean ± SEM (n = 5); **P ≤ 0.01. (B) Left: Representative cropped blot images demonstrating immunoblotting for BAX in HRMEC treated with NGF or control (PBS) under hypoxic conditions, as described in Section 2. Vinculin was used as loading control. Right: Densitometric analysis of BAX immunoblotting is shown. The protein amounts of BAX were normalized to Vinculin and are shown relative to control; the ratio BAX/Vinculin in HRMEC treated with PBS was set as one in each experiment. Data are presented as mean ± SEM (n = 6); *P ≤ 0.05

BCL2 family proteins are key regulators of mitochondria–mediated (intrinsic) apoptosis.^42^ The anti‐apoptotic proteins BCL2 and BCL‐xL preserve mitochondrial membrane integrity and prevent cytochrome c release from the mitochondria, while the pro‐apoptotic proteins BAX, BAK and BID coordinately promote pore formation in the mitochondrial membrane and induce cytochrome c leakage.^43^ Consistent with the anti‐apoptotic effect of NGF, HRMEC treatment with NGF in hypoxia increased the levels of BCL2 (Figure 3A) and decreased the levels of BAX (Figure 3B).

**Figure 4:**
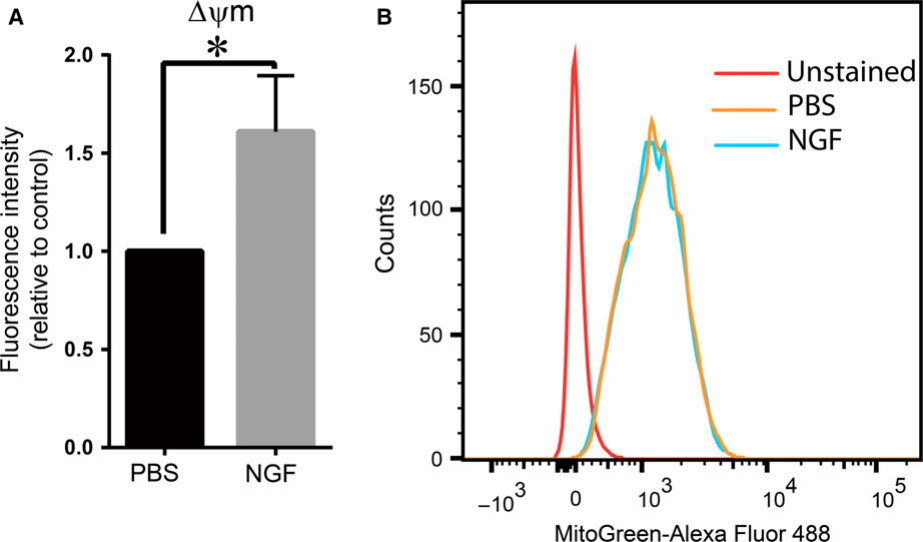
NGF preserves mitochondrial integrity in HRMEC under hypoxia. (A) HRMEC were treated with PBS or 100 ng/mL NGF under hypoxia, as described in Section 2, and were subsequently stained with TMRE, which accumulates in active mitochondria. Upon a decrease in mitochondrial potential, TMRE fails to be sequestered. The mitochondrial potential (ΔΨm) of HRMEC treated with NGF or control (PBS) under hypoxia (1% O_2_) is shown. Data are shown relative to control (PBS), which was set as 1 in each experiment. Data are presented as mean ± SEM (n = 4); *P ≤ 0.05. (B) HRMEC were treated with PBS or NGF under hypoxic conditions (1% O_2_), stained with mitoTracker Green probe for labeling mitochondria and analysed by flow cytometry. A representative histogram of cells treated with PBS and NGF is shown; similar results were observed in 3 additional experiments

Next, we questioned whether NGF affects mitochondrial membrane integrity under pro‐apoptotic conditions. To this end, HRMEC were treated under serum‐free conditions in hypoxia (1% O_2_) in the presence or absence of NGF and they were then stained with the TMRE dye. NGF increased the mitochondrial membrane potential (ΔΨm) (Figure 4A). On the contrary, NGF did not affect the mitochondrial load of the cells, as shown by Mitotracker Green staining (Figure 4B). These findings collectively indicate that NGF protected mitochondrial function and prevented starvation–induced intrinsic apoptosis under hypoxia.

### 3.3 Signaling pathways involved in the pro‐survival effect of NGF in endothelial cells under hypoxia

**Figure 5:**
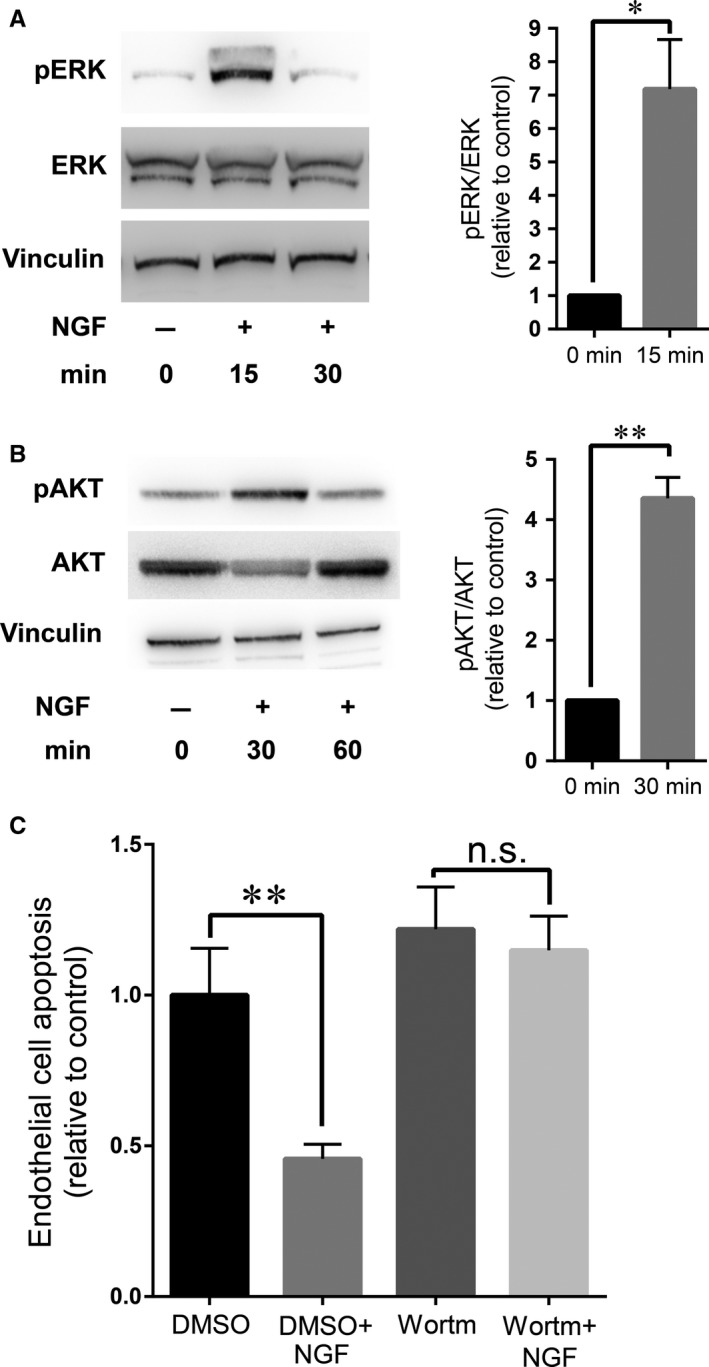
AKT mediates the anti‐apoptotic effect of NGF. (A, B) HRMEC were cultured under hypoxic conditions, treated without or with 100 ng/mL NGF and analysed at different time points, as indicated. (A) Representative cropped blot images (left) of phosphoERK, total ERK and Vinculin and the densitometric quantification of the ratio phosphoERK/ERK (right) are shown. Data are shown as relative to control (0 min); in each experiment the ratio phosphoERK/ERK of the control treatment was set as 1. Data are presented as mean ± SEM (n = 4); *P ≤ 0.05. (B) Representative cropped blot images (left) of phosphoAKT, total AKT and Vinculin and the densitometric quantification of the ratio phosphoAKT/AKT (right) are shown. Data are shown as relative to control (0 min); in each experiment the ratio phosphoAKT/AKT of the control treatment was set as 1. Data are presented as mean ± SEM (n = 6); **P ≤ 0.01. (C) HRMEC cells were pretreated with Wortmannin (an inhibitor of PI3K/AKT) or vehicle control (DMSO) followed by treatment without or with NGF under hypoxic conditions, as described in Section 2. Apoptosis of HRMEC was assessed by immunofluorescence staining for cleaved caspase‐3. Apoptosis is shown relative to control; apoptosis in control (DMSO)‐treated cells was set as 1. Data (mean ± SEM) are from one experiment performed in six replicates; similar results were observed in 3 additional experiments; **P ≤ 0.01

Next, we sought to reveal which signaling pathways mediate the anti‐apoptotic effect of NGF. We exposed HRMEC to hypoxia, treated them with NGF for different time intervals following previously reported experimental settings,^26^, ^39^, ^44^ and examined the activation of ERK and AKT pathways. NGF induces ERK and AKT phosphorylation in neuronal and EC within 60 minutes after stimulation.^26^, ^39^ NGF stimulated ERK phosphorylation at 15 minutes (Figure 5A) and AKT phosphorylation at 30 minutes (Figure 5B). Furthermore, AKT inhibition by Wortmannin abolished the anti‐apoptotic effect of NGF on HRMECs under hypoxic conditions (Figure 5C). These findings suggest that the AKT pathway is a key mediator of the anti‐apoptotic effect of NGF in EC.

## 4 DISCUSSION

Pathological neovascularization is an important feature of ROP and DR, which are leading causes of blindness in infants and adults respectively.^45^, ^46^ The role of growth factors, such as VEGF, Insulin Growth Factor or Erythropoietin, in pathological retinal vascularization has been extensively described.^47^ NGF is expressed in the developing and adult retina and is produced by retinal ganglion cells, bipolar cells, glial cells and retinal pigment epithelial cells.^48^, ^49^ Furthermore, retinas of pups subjected to the ROP model display increased NGF expression.^24^ The enhanced NGF expression could be a result of increased inflammation in the diseased retinas,^50^ as NGF expression is shown to be upregulated under inflammatory conditions.^4^, ^15^ In turn, TrkA is expressed in the outer and inner segments of the retina, in photoreceptors, bipolar cells, Müller cells, amacrine cells, ganglion cells and astrocytes,^49^ as well as in retinal EC (HRMEC).^37^ Experimental evidence from different cell systems and animal models suggested that NGF promotes angiogenesis.^17^, ^19^, ^25^ However, little was known about its role in hypoxia‐triggered pathological retinal angiogenesis.

Here, we studied the effect of NGF on pathological neovascularization in experimental ROP. Application of exogenous NGF enhanced pathological vascularization, while antibody neutralization of endogenous NGF significantly reduced it. These findings are in keeping with previous studies showing a TrkA–dependent stimulatory effect of NGF on retinal neovascularization in mice subjected to the ROP model.^24^ On the contrary, NGF did not affect revascularization of the retinal avascular area in the ROP model. Thus, NGF is predominantly involved in pathological neovascular tuft formation and may not affect the cellular processes associated with revascularization of the avascular area of the retina.^51^, ^52^

Mechanistically we showed here that the pro‐angiogenic effect of NGF is associated with reduced apoptosis rather than with enhanced proliferation of retinal EC. Furthermore, in vitro studies in HRMEC revealed that the anti‐apoptotic effect of NGF was only observed in hypoxia and not in normoxia. Although NGF was previously shown to induce angiogenesis‐related functions in retinal EC under normoxic conditions,^24^, ^41^ we found here that the anti‐apoptotic effect of NGF on EC is restricted to hypoxic conditions. Why NGF exhibits its anti‐apoptotic effect in retinal EC primarily under hypoxia is still unclear. It was previously shown that the NGF receptor p75 undergoes oxygen–dependent cleavage, which subsequently mediates HIF‐1α stabilization.^53^ Whether p75 cleavage could regulate the anti‐apoptotic effect of NGF is not known and requires further investigation. Furthermore, it is not known whether p75 cleavage could affect its association with TrkA or binding of NGF to TrkA/p75 heterodimers and whether all these functions could collectively contribute to the anti‐apoptotic effect of NGF under hypoxia. These aspects require further investigation.

In addition, we identified AKT and ERK activation to mediate the anti‐apoptotic effect of NGF in HRMEC. AKT and ERK activate the transcription factor CREB, which induces BCL2 expression.^16^, ^54^, ^55^ Consistently, we found that NGF treatment increased BCL2 expression, while it decreased BAX expression under hypoxic conditions. In keeping with these findings, NGF preserved the mitochondrial membrane potential under pro‐apoptotic conditions (starvation) in combination with hypoxia.

Hence, our findings reveal a direct pro‐survival effect of NGF in retinal EC under hypoxic conditions, which thereby contributes to elevated pathological neovascularization in retinas of mice subjected to the ROP model. The therapeutic engagement of neuroprotective agents, such as NGF, in the context of retinopathies has been recently considered.^4^, ^5^ Our findings however suggest that caution is needed in this scenario, as the pro‐angiogenic effects of NGF may act detrimentally in the course of proliferative retinopathies like DR, by worsening pathological neovascularization. Therefore, the potential administration of NGF as a neuroprotective agent in the context of DR should be monitored very closely.

## CONFLICT OF INTEREST

None.
